# Poly[potassium-μ-2-[2-(carboxymethyl)phenyl]acetato]

**DOI:** 10.1107/S1600536808038889

**Published:** 2008-11-29

**Authors:** Reyes García-Zarracino, Marcela Rangel-Marrón, Hugo Tlahuext, Herbert Höpfl

**Affiliations:** a Facultad de Química, Universidad Autónoma del Carmen. Calle 56 No. 4, CP 24180, Cd. del Carmen, Campeche, México; b Centro de Investigaciones Químicas, Universidad Autónoma del Estado de Morelos. Av. Universidad 1001 Col., Chamilpa, CP 62209, Cuernavaca Mor., México

## Abstract

In the title salt, [K(C_10_H_9_O_4_)]_*n*_, the K^+^ ions are coordinated by six O atoms from three different anions, and there is a cation–π inter­action at *ca* 3.14 Å. The 2-[2-(carboxymethyl)phenyl]acetate anions are stabilized by intramolecular O—H⋯O hydrogen bonds, and the K^+^ cations are linked into one-dimensional coordination polymers running along the *b* axis; these are further inter­connected by weak C—H⋯O hydrogen bonds.

## Related literature

For general background, see: Atwood & Steed (2004 ▶); Ma & Dougherty (1997 ▶); Kumpf & Dougherty (1993 ▶); Heginbotham *et al.* (1994 ▶). For coordination polymers, see: Chae *et al.* (2004 ▶); García-Zarracino *et al.* (2003 ▶); García-Zarracino & Höpfl (2004 ▶). For analysis of hydrogen-bonding patterns, see: Bernstein *et al.* (1995 ▶); Desiraju (2002 ▶).
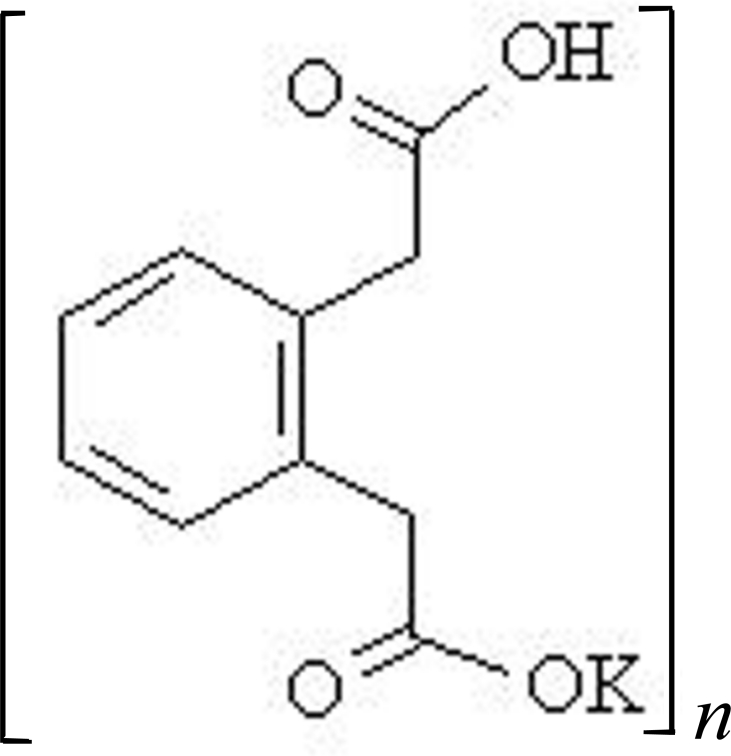


## Experimental

### 

#### Crystal data

[K(C_10_H_9_O_4_)]
*M* *_r_* = 232.27
Monoclinic, 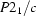

*a* = 8.3365 (14) Å
*b* = 6.7886 (11) Å
*c* = 17.651 (3) Å
β = 92.543 (3)°
*V* = 997.9 (3) Å^3^
*Z* = 4
Mo *K*α radiation
μ = 0.52 mm^−1^
*T* = 293 (2) K
0.45 × 0.30 × 0.28 mm

#### Data collection

Bruker SMART APEX CCD area-detector diffractometer
Absorption correction: multi-scan (*SADABS*; Sheldrick, 1996 ▶) *T*_min_ = 0.713, *T*_max_ = 0.864
8711 measured reflections
1727 independent reflections
1560 reflections with *I* > 2σ(*I*)
*R*_int_ = 0.039

#### Refinement

*R*[*F* ^2^ > 2σ(*F* ^2^)] = 0.041
*wR*(*F* ^2^) = 0.101
*S* = 1.08
1727 reflections
139 parameters
H atoms treated by a mixture of independent and constrained refinement
Δρ_max_ = 0.21 e Å^−3^
Δρ_min_ = −0.20 e Å^−3^

### 

Data collection: *SMART* (Bruker, 2000 ▶); cell refinement: *SAINT-Plus-NT* (Bruker, 2001 ▶); data reduction: *SAINT-Plus-NT*; program(s) used to solve structure: *SHELXTL-NT* (Sheldrick, 2008 ▶); program(s) used to refine structure: *SHELXTL-NT*; molecular graphics: *SHELXTL-NT*; software used to prepare material for publication: *PLATON* (Spek, 2003 ▶) and *publCIF* (Westrip, 2008 ▶).

## Supplementary Material

Crystal structure: contains datablocks I, global. DOI: 10.1107/S1600536808038889/sg2283sup1.cif

Structure factors: contains datablocks I. DOI: 10.1107/S1600536808038889/sg2283Isup2.hkl

Additional supplementary materials:  crystallographic information; 3D view; checkCIF report

## Figures and Tables

**Table 1:** Hydrogen-bond geometry (Å, °)

*D*—H⋯*A*	*D*—H	H⋯*A*	*D*⋯*A*	*D*—H⋯*A*
O4—H4′⋯O1^i^	1.01 (3)	2.57 (3)	3.248 (2)	125 (2)
O4—H4′⋯O2^i^	1.01 (3)	1.47 (3)	2.471 (2)	176 (3)
C2—H2*A*⋯O2^ii^	0.97	2.53	3.480 (3)	167

Symmetry codes: (i) 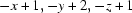; (ii) 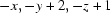.

**Fig. 1.**
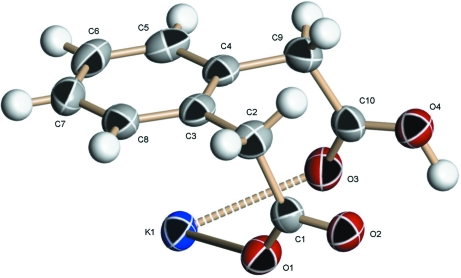
Asymmetric unit of I showing 50% probability displacement ellipsoids and the atomic numbering. H atoms are shown as small spheres of arbitrary radius.

**Fig. 2.**
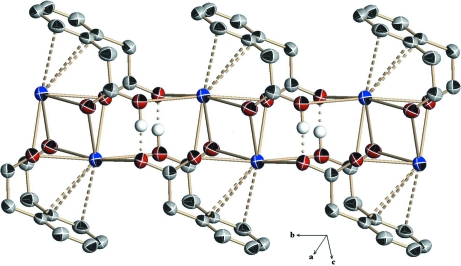
View of the one-dimensional coordination polymer chain running along to the b axis. Dashed and dotted lines indicate Caryl···K+ and O—H···O contacts, respectively. H atoms not involved in hydrogen-bonding have been omitted for clarity.

**Fig. 3.**
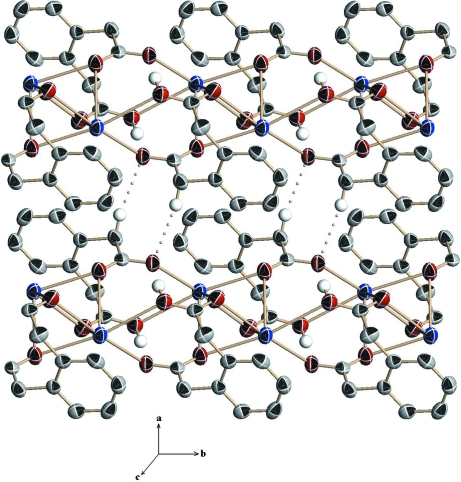
View of the C—H···O hydogen bonds forming R22(8) motifs between adjacent coordination polymers. The hydrogen bonds are represented by dotted lines and H atoms not involved in hydrogen- bonding have been omitted for clarity.

## supplementary crystallographic information

### Comment

Hydrogen bonding, cation-π, and π-π interactions are principal forces which determine the structure, self-assembly and recognition in many chemical and biological systems (Atwood & Steed, 2004). Cation-π interactions are now recognized as important non-covalent binding forces in biological systems (Ma & Dougherty, 1997). It has been postulated that the aromatic side chains of amino acids might determine K^+ ^transport selectivity in transmembrane protein channels (Kumpf & Dougherty, 1993; Heginbotham *et al.*, 1994).

In complex I each K^+ ^ion is coordinated by six oxygen atoms from three different ligand molecules and there is a cation···π interaction (Fig.1) forming a distorted square-face monocapped prism (Fig. 2). The centroid of the aryl ring (*Cg*, C3—C8) is situated 3.138 Å from the K^+^ ion [sum of ionic and van der Waals radii K^+^···C(Ar) 3.37 Å]. Since the K—C_arene_ contacts [C3—K= 3.344 (2), C4—K = 3.260 (2) and C5—K = 3.334 (2) Å] and [C6—K = 3.508 (2), C7—K = 3.596, C8—K = 3.511 (2)] the K-arene interaction can be regarded as η^3^-coordination. The oxygen atoms of the carboxyl and carboxylate groups are forming bridging units between two K^+^ cations, thus generating a one-dimensional coordination polymer, running along the *b* axis (Chae *et al.*, 2004; García-Zarracino *et al.*, 2003; García-Zarracino & Höpfl, 2004) (Fig. 2). The coordination polymer is stabilized by intramolecular O4—H4'···O2 hydrogen bonds. The crystal structure is stabilized by weak C—H···O hydogen bonds forming *R*_2_^2^(8) motifs, (Bernstein *et al.*, 1995; Desiraju, 2002) between adjacent coordination polymers (Fig. 3, Table 1).

### Experimental

Single Crystals of (I) were obtained by slow evaporation of a solution containning 1,2-phenylenediacetic acid (1.0 g, 5.15 mmol), potassium hydroxide (0.289 g, 5.15 mmol) in MeOH/H_2_O (5:1).

### Refinement

Aromatic and methylene H atoms were positioned geometrically and constrained using the riding-model approximation [*C*-Haryl = 0.93 Å, *U*_iso_(H_aryl_)= 1.2 *U*_eq_(*C*_aryl_); *C*-H_methylene_ = 0.97 Å, *U*_iso_(H_methylene_) = 1.5 *U*_eq_(*C*_methylene_)]. Atom H4', which is involved in a hydrogen-bonding interaction, was located by difference Fourier map, constrained using the riding-model approximation [*U*_iso_(*O*4-H') = 1.5 *U*_eq_(O4)] and the coordinates were refined freely.

### Crystal data

**Table:**

[K(C_10_H_9_O_4_)]	*F*_000_ = 480
*M**_r_* = 232.27	*D*_x_ = 1.546 Mg m^−^^3^
Monoclinic, *P*2_1_/*c*	Mo *K*α radiation λ = 0.71073 Å
Hall symbol: -P 2ybc	Cell parameters from 4833 reflections
*a* = 8.3365 (14) Å	θ = 2.3–28.2º
*b* = 6.7886 (11) Å	µ = 0.52 mm^−^^1^
*c* = 17.651 (3) Å	*T* = 293 (2) K
β = 92.543 (3)º	Rectangular, colourless
*V* = 997.9 (3) Å^3^	0.45 × 0.30 × 0.28 mm
*Z* = 4	

### Data collection

**Table:**

Bruker SMART APEX CCD area-detector diffractometer	1727 independent reflections
Radiation source: fine-focus sealed tube	1560 reflections with *I* > 2σ(*I*)
Monochromator: graphite	*R*_int_ = 0.039
*T* = 293(2) K	θ_max_ = 25.0º
φ and ω scans	θ_min_ = 2.3º
Absorption correction: multi-scan(SADABS; Sheldrick, 1996)	*h* = −9→9
*T*_min_ = 0.713, *T*_max_ = 0.864	*k* = −8→8
8711 measured reflections	*l* = −20→20

### Refinement

**Table:**

Refinement on *F*^2^	Secondary atom site location: difference Fourier map
Least-squares matrix: full	Hydrogen site location: inferred from neighbouring sites
*R*[*F*^2^ > 2σ(*F*^2^)] = 0.041	H atoms treated by a mixture of independent and constrained refinement
*wR*(*F*^2^) = 0.101	*w* = 1/[σ^2^(*F*_o_^2^) + (0.0462*P*)^2^ + 0.3852*P*] where *P* = (*F*_o_^2^ + 2*F*_c_^2^)/3
*S* = 1.08	(Δ/σ)_max_ = 0.001
1727 reflections	Δρ_max_ = 0.21 e Å^−^^3^
139 parameters	Δρ_min_ = −0.20 e Å^−^^3^
Primary atom site location: structure-invariant direct methods	Extinction correction: none

### Special details

**Table:**

Geometry. All e.s.d.'s (except the e.s.d. in the dihedral angle between two l.s. planes) are estimated using the full covariance matrix. The cell e.s.d.'s are taken into account individually in the estimation of e.s.d.'s in distances, angles and torsion angles; correlations between e.s.d.'s in cell parameters are only used when they are defined by crystal symmetry. An approximate (isotropic) treatment of cell e.s.d.'s is used for estimating e.s.d.'s involving l.s. planes.
Refinement. Refinement of *F*^2^ against ALL reflections. The weighted *R*-factor *wR* and goodness of fit *S* are based on *F*^2^, conventional *R*-factors *R* are based on *F*, with *F* set to zero for negative *F*^2^. The threshold expression of *F*^2^ > σ(*F*^2^) is used only for calculating *R*-factors(gt) *etc*. and is not relevant to the choice of reflections for refinement. *R*-factors based on *F*^2^ are statistically about twice as large as those based on *F*, and *R*- factors based on ALL data will be even larger.

### Fractional atomic coordinates and isotropic or equivalent isotropic displacement parameters (Å^2^)

**Table:**

	*x*	*y*	*z*	*U*_iso_*/*U*_eq_	
K1	0.38073 (6)	0.33470 (7)	0.44502 (3)	0.0455 (2)	
O1	0.2998 (2)	0.6914 (2)	0.51210 (9)	0.0487 (4)	
O2	0.26242 (19)	1.0124 (2)	0.52043 (9)	0.0449 (4)	
O3	0.5412 (2)	0.6608 (2)	0.38998 (11)	0.0569 (5)	
O4	0.5243 (2)	0.9834 (2)	0.37776 (9)	0.0471 (4)	
H4'	0.612 (4)	0.991 (4)	0.4186 (16)	0.071*	
C1	0.2360 (3)	0.8431 (3)	0.49014 (12)	0.0350 (5)	
C2	0.1154 (3)	0.8450 (3)	0.42353 (13)	0.0449 (6)	
H2A	0.0091	0.8637	0.4426	0.054*	
H2B	0.1379	0.9572	0.3917	0.054*	
C3	0.1135 (3)	0.6626 (3)	0.37550 (12)	0.0362 (5)	
C4	0.2223 (3)	0.6353 (3)	0.31858 (12)	0.0378 (5)	
C5	0.2138 (3)	0.4620 (4)	0.27695 (12)	0.0458 (6)	
H5	0.2859	0.4419	0.2389	0.055*	
C6	0.1017 (3)	0.3192 (4)	0.29041 (14)	0.0495 (6)	
H6	0.0988	0.2039	0.2619	0.059*	
C7	−0.0052 (3)	0.3471 (3)	0.34570 (14)	0.0498 (6)	
H7	−0.0816	0.2512	0.3549	0.060*	
C8	0.0006 (3)	0.5179 (3)	0.38770 (13)	0.0434 (6)	
H8	−0.0730	0.5365	0.4251	0.052*	
C9	0.3453 (3)	0.7892 (4)	0.30161 (13)	0.0497 (6)	
H9A	0.2919	0.9158	0.2963	0.060*	
H9B	0.3909	0.7583	0.2534	0.060*	
C10	0.4803 (3)	0.8065 (3)	0.36163 (12)	0.0393 (5)	

### Atomic displacement parameters (Å^2^)

**Table:**

	*U*^11^	*U*^22^	*U*^33^	*U*^12^	*U*^13^	*U*^23^
K1	0.0465 (3)	0.0342 (3)	0.0548 (3)	0.0001 (2)	−0.0082 (2)	0.0024 (2)
O1	0.0540 (10)	0.0367 (9)	0.0542 (10)	0.0060 (8)	−0.0139 (8)	−0.0016 (7)
O2	0.0498 (10)	0.0346 (8)	0.0497 (9)	0.0011 (7)	−0.0047 (7)	−0.0062 (7)
O3	0.0503 (10)	0.0450 (10)	0.0735 (12)	−0.0046 (8)	−0.0172 (9)	0.0114 (9)
O4	0.0466 (10)	0.0432 (9)	0.0508 (9)	−0.0003 (7)	−0.0053 (8)	0.0004 (7)
C1	0.0315 (11)	0.0362 (12)	0.0379 (11)	−0.0003 (9)	0.0064 (9)	0.0003 (9)
C2	0.0455 (13)	0.0393 (13)	0.0492 (13)	0.0099 (10)	−0.0077 (11)	−0.0032 (10)
C3	0.0352 (11)	0.0354 (11)	0.0372 (11)	0.0058 (9)	−0.0090 (9)	−0.0001 (9)
C4	0.0335 (11)	0.0442 (12)	0.0350 (11)	0.0027 (9)	−0.0077 (9)	0.0037 (9)
C5	0.0416 (13)	0.0596 (15)	0.0357 (12)	0.0124 (11)	−0.0051 (10)	−0.0076 (11)
C6	0.0541 (15)	0.0428 (14)	0.0498 (14)	0.0022 (11)	−0.0170 (12)	−0.0106 (11)
C7	0.0502 (14)	0.0435 (13)	0.0542 (15)	−0.0105 (11)	−0.0139 (12)	0.0053 (11)
C8	0.0387 (13)	0.0508 (14)	0.0405 (12)	−0.0034 (10)	−0.0013 (10)	0.0031 (10)
C9	0.0455 (14)	0.0583 (15)	0.0446 (13)	−0.0076 (12)	−0.0052 (11)	0.0133 (11)
C10	0.0339 (12)	0.0448 (14)	0.0395 (12)	−0.0037 (10)	0.0056 (9)	0.0053 (10)

### Geometric parameters (Å, °)

**Table:**

K1—O1^i^	2.7422 (17)	O4—H4'	1.00 (3)
K1—O2^ii^	2.7654 (17)	C1—C2	1.513 (3)
K1—O3	2.7837 (18)	C2—C3	1.500 (3)
K1—O1	2.7912 (16)	C2—H2A	0.9700
K1—O4^ii^	2.9419 (17)	C2—H2B	0.9700
K1—O3^i^	2.956 (2)	C3—C8	1.384 (3)
K1—C4	3.260 (2)	C3—C4	1.396 (3)
K1—C5	3.334 (2)	C4—C5	1.387 (3)
K1—C3	3.344 (2)	C4—C9	1.503 (3)
K1—C6	3.508 (2)	C5—C6	1.374 (3)
K1—C8	3.511 (2)	C5—H5	0.9300
K1—K1^i^	3.5235 (10)	C6—C7	1.364 (4)
O1—C1	1.215 (2)	C6—H6	0.9300
O1—K1^i^	2.7422 (17)	C7—C8	1.376 (3)
O2—C1	1.282 (2)	C7—H7	0.9300
O2—K1^iii^	2.7654 (17)	C8—H8	0.9300
O3—C10	1.210 (3)	C9—C10	1.515 (3)
O3—K1^i^	2.956 (2)	C9—H9A	0.9700
O4—C10	1.284 (3)	C9—H9B	0.9700
O4—K1^iii^	2.9420 (17)		
			
O1^i^—K1—O2^ii^	100.31 (5)	C8—K1—K1^i^	114.41 (4)
O1^i^—K1—O3	70.84 (5)	C1—O1—K1^i^	123.28 (14)
O2^ii^—K1—O3	169.83 (5)	C1—O1—K1	135.31 (14)
O1^i^—K1—O1	100.90 (5)	K1^i^—O1—K1	79.10 (5)
O2^ii^—K1—O1	112.58 (5)	C1—O2—K1^iii^	124.52 (13)
O3—K1—O1	65.62 (6)	C10—O3—K1	126.51 (15)
O1^i^—K1—O4^ii^	69.61 (5)	C10—O3—K1^i^	117.98 (15)
O2^ii^—K1—O4^ii^	73.33 (5)	K1—O3—K1^i^	75.67 (5)
O3—K1—O4^ii^	107.07 (6)	C10—O4—K1^iii^	136.90 (14)
O1—K1—O4^ii^	169.97 (5)	C10—O4—H4'	113.6 (16)
O1^i^—K1—O3^i^	63.90 (5)	K1^iii^—O4—H4'	87.9 (16)
O2^ii^—K1—O3^i^	66.52 (5)	O1—C1—O2	124.2 (2)
O3—K1—O3^i^	104.32 (5)	O1—C1—C2	121.41 (19)
O1—K1—O3^i^	67.66 (5)	O2—C1—C2	114.38 (18)
O4^ii^—K1—O3^i^	109.28 (5)	C3—C2—C1	114.97 (18)
O1^i^—K1—C4	126.04 (6)	C3—C2—H2A	108.5
O2^ii^—K1—C4	133.04 (5)	C1—C2—H2A	108.5
O3—K1—C4	56.60 (5)	C3—C2—H2B	108.5
O1—K1—C4	69.46 (5)	C1—C2—H2B	108.5
O4^ii^—K1—C4	113.01 (5)	H2A—C2—H2B	107.5
O3^i^—K1—C4	137.12 (5)	C8—C3—C4	119.1 (2)
O1^i^—K1—C5	128.62 (6)	C8—C3—C2	119.2 (2)
O2^ii^—K1—C5	119.32 (6)	C4—C3—C2	121.7 (2)
O3—K1—C5	70.82 (6)	C8—C3—K1	85.25 (13)
O1—K1—C5	93.06 (5)	C4—C3—K1	74.46 (11)
O4^ii^—K1—C5	90.70 (5)	C2—C3—K1	110.62 (12)
O3^i^—K1—C5	159.86 (6)	C5—C4—C3	118.3 (2)
C4—K1—C5	24.26 (6)	C5—C4—C9	120.2 (2)
O1^i^—K1—C3	140.12 (5)	C3—C4—C9	121.5 (2)
O2^ii^—K1—C3	117.21 (5)	C5—C4—K1	80.87 (12)
O3—K1—C3	70.43 (5)	C3—C4—K1	81.18 (12)
O1—K1—C3	53.74 (5)	C9—C4—K1	108.22 (13)
O4^ii^—K1—C3	131.90 (5)	C6—C5—C4	121.7 (2)
O3^i^—K1—C3	117.95 (5)	C6—C5—K1	85.62 (14)
C4—K1—C3	24.36 (5)	C4—C5—K1	74.87 (12)
C5—K1—C3	41.93 (5)	C6—C5—H5	119.1
O1^i^—K1—C6	144.60 (6)	C4—C5—H5	119.1
O2^ii^—K1—C6	96.45 (6)	K1—C5—H5	110.3
O3—K1—C6	93.71 (6)	C7—C6—C5	119.8 (2)
O1—K1—C6	101.04 (5)	C7—C6—K1	82.60 (14)
O4^ii^—K1—C6	85.95 (5)	C5—C6—K1	71.39 (13)
O3^i^—K1—C6	151.19 (6)	C7—C6—H6	120.1
C4—K1—C6	41.56 (6)	C5—C6—H6	120.1
C5—K1—C6	23.00 (6)	K1—C6—H6	116.5
C3—K1—C6	47.66 (5)	C6—C7—C8	119.6 (2)
O1^i^—K1—C8	162.86 (5)	C6—C7—K1	75.30 (14)
O2^ii^—K1—C8	94.72 (5)	C8—C7—K1	75.38 (13)
O3—K1—C8	93.40 (5)	C6—C7—H7	120.2
O1—K1—C8	65.34 (5)	C8—C7—H7	120.2
O4^ii^—K1—C8	123.29 (5)	K1—C7—H7	120.2
O3^i^—K1—C8	115.89 (5)	C7—C8—C3	121.5 (2)
C4—K1—C8	41.24 (5)	C7—C8—K1	82.34 (14)
C5—K1—C8	46.86 (6)	C3—C8—K1	71.62 (12)
C3—K1—C8	23.13 (5)	C7—C8—H8	119.3
C6—K1—C8	39.43 (6)	C3—C8—H8	119.3
O1^i^—K1—K1^i^	51.07 (3)	K1—C8—H8	117.3
O2^ii^—K1—K1^i^	116.32 (4)	C4—C9—C10	113.96 (18)
O3—K1—K1^i^	54.37 (4)	C4—C9—H9A	108.8
O1—K1—K1^i^	49.84 (4)	C10—C9—H9A	108.8
O4^ii^—K1—K1^i^	120.59 (4)	C4—C9—H9B	108.8
O3^i^—K1—K1^i^	49.95 (3)	C10—C9—H9B	108.8
C4—K1—K1^i^	100.37 (4)	H9A—C9—H9B	107.7
C5—K1—K1^i^	121.87 (5)	O3—C10—O4	124.2 (2)
C3—K1—K1^i^	97.39 (4)	O3—C10—C9	120.7 (2)
C6—K1—K1^i^	141.83 (5)	O4—C10—C9	115.0 (2)
			
O1^i^—K1—O1—C1	126.9 (2)	C3—K1—C4—C9	120.3 (2)
O2^ii^—K1—O1—C1	−127.0 (2)	C6—K1—C4—C9	−147.96 (19)
O3—K1—O1—C1	63.9 (2)	C8—K1—C4—C9	150.69 (19)
O4^ii^—K1—O1—C1	108.4 (3)	K1^i^—K1—C4—C9	35.31 (16)
O3^i^—K1—O1—C1	−177.4 (2)	C3—C4—C5—C6	0.2 (3)
C4—K1—O1—C1	2.3 (2)	C9—C4—C5—C6	−179.2 (2)
C5—K1—O1—C1	−3.4 (2)	K1—C4—C5—C6	74.9 (2)
C3—K1—O1—C1	−19.0 (2)	C3—C4—C5—K1	−74.75 (17)
C6—K1—O1—C1	−25.2 (2)	C9—C4—C5—K1	105.83 (18)
C8—K1—O1—C1	−42.4 (2)	O1^i^—K1—C5—C6	142.02 (14)
K1^i^—K1—O1—C1	126.9 (2)	O2^ii^—K1—C5—C6	6.69 (16)
O1^i^—K1—O1—K1^i^	0.0	O3—K1—C5—C6	−174.26 (16)
O2^ii^—K1—O1—K1^i^	106.11 (5)	O1—K1—C5—C6	−111.41 (15)
O3—K1—O1—K1^i^	−62.98 (5)	O4^ii^—K1—C5—C6	77.89 (14)
O4^ii^—K1—O1—K1^i^	−18.4 (3)	O3^i^—K1—C5—C6	−95.1 (2)
O3^i^—K1—O1—K1^i^	55.70 (5)	C4—K1—C5—C6	−124.5 (2)
C4—K1—O1—K1^i^	−124.58 (6)	C3—K1—C5—C6	−92.50 (16)
C5—K1—O1—K1^i^	−130.30 (6)	C8—K1—C5—C6	−59.92 (14)
C3—K1—O1—K1^i^	−145.89 (8)	K1^i^—K1—C5—C6	−154.75 (13)
C6—K1—O1—K1^i^	−152.05 (6)	O1^i^—K1—C5—C4	−93.44 (14)
C8—K1—O1—K1^i^	−169.22 (6)	O2^ii^—K1—C5—C4	131.23 (13)
O1^i^—K1—O3—C10	−169.7 (2)	O3—K1—C5—C4	−49.72 (14)
O2^ii^—K1—O3—C10	−139.5 (3)	O1—K1—C5—C4	13.13 (14)
O1—K1—O3—C10	−57.54 (19)	O4^ii^—K1—C5—C4	−157.57 (14)
O4^ii^—K1—O3—C10	129.80 (19)	O3^i^—K1—C5—C4	29.4 (3)
O3^i^—K1—O3—C10	−114.4 (2)	C3—K1—C5—C4	32.04 (12)
C4—K1—O3—C10	23.11 (18)	C6—K1—C5—C4	124.5 (2)
C5—K1—O3—C10	45.16 (19)	C8—K1—C5—C4	64.62 (13)
C3—K1—O3—C10	0.58 (19)	K1^i^—K1—C5—C4	−30.21 (15)
C6—K1—O3—C10	42.9 (2)	C4—C5—C6—C7	0.3 (3)
C8—K1—O3—C10	3.4 (2)	K1—C5—C6—C7	69.5 (2)
K1^i^—K1—O3—C10	−114.4 (2)	C4—C5—C6—K1	−69.20 (19)
O1^i^—K1—O3—K1^i^	−55.29 (4)	O1^i^—K1—C6—C7	178.97 (13)
O2^ii^—K1—O3—K1^i^	−25.1 (3)	O2^ii^—K1—C6—C7	60.94 (15)
O1—K1—O3—K1^i^	56.88 (4)	O3—K1—C6—C7	−119.50 (15)
O4^ii^—K1—O3—K1^i^	−115.78 (5)	O1—K1—C6—C7	−53.63 (15)
O3^i^—K1—O3—K1^i^	0.0	O4^ii^—K1—C6—C7	133.63 (15)
C4—K1—O3—K1^i^	137.53 (7)	O3^i^—K1—C6—C7	9.7 (2)
C5—K1—O3—K1^i^	159.58 (6)	C4—K1—C6—C7	−94.26 (16)
C3—K1—O3—K1^i^	115.00 (6)	C5—K1—C6—C7	−124.9 (2)
C6—K1—O3—K1^i^	157.33 (5)	C3—K1—C6—C7	−60.36 (14)
C8—K1—O3—K1^i^	117.82 (5)	C8—K1—C6—C7	−28.65 (13)
K1^i^—O1—C1—O2	−42.1 (3)	K1^i^—K1—C6—C7	−89.04 (16)
K1—O1—C1—O2	−152.13 (16)	O1^i^—K1—C6—C5	−56.10 (18)
K1^i^—O1—C1—C2	138.33 (17)	O2^ii^—K1—C6—C5	−174.13 (14)
K1—O1—C1—C2	28.3 (3)	O3—K1—C6—C5	5.43 (15)
K1^iii^—O2—C1—O1	120.5 (2)	O1—K1—C6—C5	71.30 (15)
K1^iii^—O2—C1—C2	−60.0 (2)	O4^ii^—K1—C6—C5	−101.44 (14)
O1—C1—C2—C3	−15.3 (3)	O3^i^—K1—C6—C5	134.65 (15)
O2—C1—C2—C3	165.1 (2)	C4—K1—C6—C5	30.67 (13)
C1—C2—C3—C8	97.3 (2)	C3—K1—C6—C5	64.57 (14)
C1—C2—C3—C4	−83.0 (3)	C8—K1—C6—C5	96.28 (16)
C1—C2—C3—K1	1.1 (2)	K1^i^—K1—C6—C5	35.89 (18)
O1^i^—K1—C3—C8	−172.79 (12)	C5—C6—C7—C8	−0.2 (3)
O2^ii^—K1—C3—C8	−14.49 (15)	K1—C6—C7—C8	63.3 (2)
O3—K1—C3—C8	172.82 (15)	C5—C6—C7—K1	−63.6 (2)
O1—K1—C3—C8	−113.60 (15)	O1^i^—K1—C7—C6	−2.6 (3)
O4^ii^—K1—C3—C8	77.11 (15)	O2^ii^—K1—C7—C6	−119.40 (15)
O3^i^—K1—C3—C8	−90.94 (13)	O3—K1—C7—C6	63.61 (15)
C4—K1—C3—C8	121.97 (19)	O1—K1—C7—C6	127.72 (15)
C5—K1—C3—C8	90.07 (15)	O4^ii^—K1—C7—C6	−47.38 (15)
C6—K1—C3—C8	58.20 (14)	O3^i^—K1—C7—C6	−173.97 (13)
K1^i^—K1—C3—C8	−139.20 (13)	C4—K1—C7—C6	63.69 (15)
O1^i^—K1—C3—C4	65.24 (15)	C5—K1—C7—C6	30.06 (14)
O2^ii^—K1—C3—C4	−136.46 (12)	C3—K1—C7—C6	97.57 (17)
O3—K1—C3—C4	50.85 (12)	C8—K1—C7—C6	126.6 (2)
O1—K1—C3—C4	124.43 (14)	K1^i^—K1—C7—C6	116.29 (14)
O4^ii^—K1—C3—C4	−44.86 (15)	O1^i^—K1—C7—C8	−129.2 (2)
O3^i^—K1—C3—C4	147.09 (12)	O2^ii^—K1—C7—C8	114.02 (14)
C5—K1—C3—C4	−31.90 (12)	O3—K1—C7—C8	−62.96 (15)
C6—K1—C3—C4	−63.77 (13)	O1—K1—C7—C8	1.15 (14)
C8—K1—C3—C4	−121.97 (19)	O4^ii^—K1—C7—C8	−173.96 (14)
K1^i^—K1—C3—C4	98.82 (12)	O3^i^—K1—C7—C8	59.46 (16)
O1^i^—K1—C3—C2	−53.34 (19)	C4—K1—C7—C8	−62.89 (14)
O2^ii^—K1—C3—C2	104.95 (15)	C5—K1—C7—C8	−96.52 (16)
O3—K1—C3—C2	−67.73 (15)	C3—K1—C7—C8	−29.00 (13)
O1—K1—C3—C2	5.85 (14)	C6—K1—C7—C8	−126.6 (2)
O4^ii^—K1—C3—C2	−163.45 (14)	K1^i^—K1—C7—C8	−10.29 (18)
O3^i^—K1—C3—C2	28.50 (17)	C6—C7—C8—C3	−0.4 (3)
C4—K1—C3—C2	−118.6 (2)	K1—C7—C8—C3	62.90 (19)
C5—K1—C3—C2	−150.48 (19)	C6—C7—C8—K1	−63.3 (2)
C6—K1—C3—C2	177.65 (19)	C4—C3—C8—C7	0.9 (3)
C8—K1—C3—C2	119.4 (2)	C2—C3—C8—C7	−179.4 (2)
K1^i^—K1—C3—C2	−19.76 (16)	K1—C3—C8—C7	−68.4 (2)
C8—C3—C4—C5	−0.8 (3)	C4—C3—C8—K1	69.28 (17)
C2—C3—C4—C5	179.52 (19)	C2—C3—C8—K1	−111.00 (18)
K1—C3—C4—C5	74.57 (17)	O1^i^—K1—C8—C7	142.7 (2)
C8—C3—C4—C9	178.6 (2)	O2^ii^—K1—C8—C7	−66.03 (14)
C2—C3—C4—C9	−1.1 (3)	O3—K1—C8—C7	120.10 (14)
K1—C3—C4—C9	−106.02 (19)	O1—K1—C8—C7	−178.74 (15)
C8—C3—C4—K1	−75.34 (18)	O4^ii^—K1—C8—C7	7.10 (16)
C2—C3—C4—K1	104.94 (18)	O3^i^—K1—C8—C7	−132.18 (14)
O1^i^—K1—C4—C5	105.32 (14)	C4—K1—C8—C7	94.82 (16)
O2^ii^—K1—C4—C5	−63.79 (16)	C5—K1—C8—C7	60.55 (15)
O3—K1—C4—C5	120.34 (15)	C3—K1—C8—C7	126.9 (2)
O1—K1—C4—C5	−165.98 (15)	C6—K1—C8—C7	28.40 (14)
O4^ii^—K1—C4—C5	24.49 (15)	K1^i^—K1—C8—C7	172.23 (13)
O3^i^—K1—C4—C5	−165.60 (13)	O1^i^—K1—C8—C3	15.9 (3)
C3—K1—C4—C5	−120.7 (2)	O2^ii^—K1—C8—C3	167.10 (13)
C6—K1—C4—C5	−29.02 (13)	O3—K1—C8—C3	−6.77 (14)
C8—K1—C4—C5	−90.37 (15)	O1—K1—C8—C3	54.39 (13)
K1^i^—K1—C4—C5	154.26 (13)	O4^ii^—K1—C8—C3	−119.77 (13)
O1^i^—K1—C4—C3	−133.94 (12)	O3^i^—K1—C8—C3	100.94 (13)
O2^ii^—K1—C4—C3	56.94 (14)	C4—K1—C8—C3	−32.06 (12)
O3—K1—C4—C3	−118.93 (14)	C5—K1—C8—C3	−66.32 (14)
O1—K1—C4—C3	−45.25 (12)	C6—K1—C8—C3	−98.48 (16)
O4^ii^—K1—C4—C3	145.22 (12)	K1^i^—K1—C8—C3	45.36 (14)
O3^i^—K1—C4—C3	−44.87 (15)	C5—C4—C9—C10	−108.5 (2)
C5—K1—C4—C3	120.7 (2)	C3—C4—C9—C10	72.1 (3)
C6—K1—C4—C3	91.71 (14)	K1—C4—C9—C10	−18.6 (2)
C8—K1—C4—C3	30.37 (11)	K1—O3—C10—O4	134.88 (18)
K1^i^—K1—C4—C3	−85.01 (12)	K1^i^—O3—C10—O4	42.3 (3)
O1^i^—K1—C4—C9	−13.62 (18)	K1—O3—C10—C9	−46.6 (3)
O2^ii^—K1—C4—C9	177.27 (14)	K1^i^—O3—C10—C9	−139.12 (17)
O3—K1—C4—C9	1.40 (14)	K1^iii^—O4—C10—O3	−116.9 (2)
O1—K1—C4—C9	75.08 (15)	K1^iii^—O4—C10—C9	64.4 (3)
O4^ii^—K1—C4—C9	−94.45 (16)	C4—C9—C10—O3	42.3 (3)
O3^i^—K1—C4—C9	75.46 (17)	C4—C9—C10—O4	−139.0 (2)
C5—K1—C4—C9	−118.9 (2)		

Symmetry codes: (i) −*x*+1, −*y*+1, −*z*+1; (ii) *x*, *y*−1, *z*; (iii) *x*, *y*+1, *z*.

### Hydrogen-bond geometry (Å, °)

**Table:**

*D*—H···*A*	*D*—H	H···*A*	*D*···*A*	*D*—H···*A*
O4—H4'···O1^iv^	1.01 (3)	2.57 (3)	3.248 (2)	125 (2)
O4—H4'···O2^iv^	1.01 (3)	1.47 (3)	2.471 (2)	176 (3)
C2—H2A···O2^v^	0.97	2.53	3.480 (3)	167

Symmetry codes: (iv) −*x*+1, −*y*+2, −*z*+1; (v) −*x*, −*y*+2, −*z*+1.
